# Optimization of extraction of loquat flowers polyphenolics and its antioxidant and anti-polyphenol oxidase properties

**DOI:** 10.1080/21655979.2020.1735604

**Published:** 2020-03-03

**Authors:** Gui-Li Huang, Jia-Jia Ma, Si-Yao Sui, Yu-Ning Wang

**Affiliations:** Agricultural Product Storage and Processing Laboratory, Suzhou Academy of Agricultural Sciences, Suzhou, China

**Keywords:** Response surface methodology, antioxidants, purified loquat flowers polyphenolics, polyphenol oxidase, molecular docking

## Abstract

In this study, the conditions of extraction of loquat flowers polyphenolics were optimized through response surface methodology (RSM). Proper extraction conditions were: solid to liquid ratio 1 g per 50 mL and ethanol concentration 50% at 61°C for 9 min. Furthermore, the antioxidant and anti-polyphenol oxidase (PPO) activity of purified total polyphenolics (PTP) were investigated. PTP displayed strong antioxidant activity with *IC*_50_ values of 126.3 ± 8.9, 162.4 ± 6.3 and 94.97 mg ascorbic acid equivalent/g dry weight (mg AAE/d.w.) for ABTS, DPPH, and FRAP assays. In addition, PTP has a substantial inhibitory activity on PPO (*IC*_50_ = 115 ± 9.2 μg/mL). From the kinetics analysis, it was proved to be a reversible and mixed-type inhibitor of PPO with *K*_I_ and *K*_IS_ values of 76.77 μg/mL and 227.86 μg/mL, respectively. Further, the molecular mechanism underlying the inhibition of PPO by PTP was investigated by molecular docking techniques. The results showed that PTP units could form interaction with the catalytic pocket of PPO through the interaction with amino acid residues in the enzyme active center. The antioxidant activities of PTP together with its effect on PPO activity provide a strong starting point for their practical usage in the food industry.

## Graphical Abstract

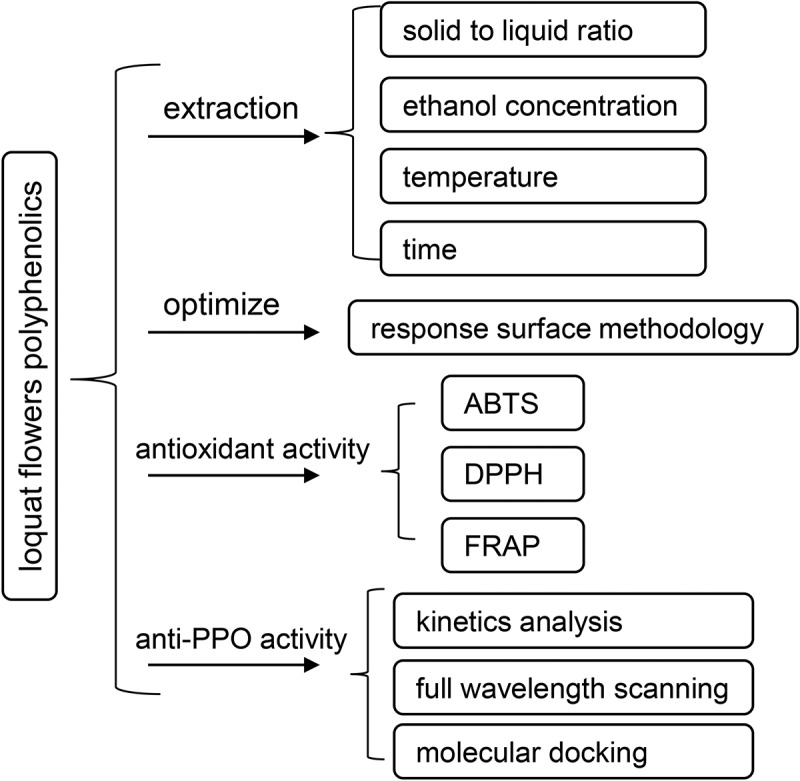

## 1. Introduction

Polyphenols as secondary metabolites of plants are omnipresent in the plant kingdom, and high contents of polyphenols have been found in foods and vegetables, such as olive, grape, blueberry, mango, sweetsop, and citrus fruits [1,2]. Polyphenols have received extensive attention because they might exert many beneficial biological properties like antioxidant, anti-aging, anti–inflammatory, cardioprotective, anticancer, and antimicrobial activity [3]. Polyphenols from tea are believed to exert their chemopreventive/chemotherapeutic action toward several types of disorders including cancer, diabetes and cardiovascular diseases through regulation of various receptor tyrosine kinases, signal transduction pathways and metastasis [4–6]. Curcumin, a polyphenolic, has shown the capacity to reduce the free radicals, and has health benefits because of its antioxidant, immune regulatory, anti–inflammatory, antidiabetic, neuroprotective, cardioprotective, anticancer, and hepatoprotective effects [7,8]. Therefore, there is an increasing attention in searching for plant polyphenols for the food and nutrition industry.

Browning of fruits and vegetables is a common undesirable phenomenon in the process of storage, which often leads to a decline of nutritional quality and economic values. Browning of fruits and vegetables has mainly given first place to enzymatic browning. Polyphenol oxidase (PPO), also known as tyrosinase, is widely existing in plants, animals, and microorganisms [9] and catalyzes the browning process in fruits and vegetables. PPO is a metalloenzyme containing two Cu^2+^ in the active site, which catalytically oxidizes polyphenols to their quinone derivates, which are further converted to melanin [10]. This encouraged researchers and scientists to explore new potent PPO inhibitors. A huge number of natural and synthetic inhibitors have already been described. Plant polyphenols, like proanthocyanidins, have been reported to exhibit inhibition of PPO activities [11,12]. However, the activity of loquat flower polyphenol toward PPO is still unclear.

Loquat (*Eriobotrya japonica* Lindl.) medicinally highly valuable fruit, grown in the subtropical area and used in traditional Chinese medicine for centuries. Loquat extracts were used for the treatment of inflammation, diabetes, chronic bronchitis, and cancer [13]. In order to achieve better the quality and yield of loquat fruit, a huge number of loquat flowers need to be removed during the planting process, which provides plenty of raw materials for the utilization of loquat flowers. However, there is a little relevant report about the loquat flower polyphenolics.

In this study, we firstly optimize the extraction conditions of polyphenolics using single-factor experiments and response surface methodology (RSM) simultaneously. Secondly, explore antioxidant activities of loquat flower polyphenolic by ABTS, DPPH, and FRAP assays. Finally, anti-PPO activity, as well as mechanism of enzyme inhibition, was studied in order to elucidate parameters important for the development of natural PPO inhibitors. This research, therefore, aimed to study the antioxidant and anti-PPO activities of PTP and provided a scientific foundation for their uses in the food industry.

## 2. Materials and methods

### 2.1 Chemicals and materials

L-DOPA (L-3,4-dihydroxyphenylalanine), vitamin C (ascorbic acid), ABTS (2,20-azino-bis (3-ethylbenzothiazoline-6-sulfonic acid)), TPTZ (2,4,6-tripyridyl-S-triazine), DPPH (2,2-diphenyl-1-picrylhydrazyl), and GA (gallic acid) were from Sigma-Aldrich (USA). FRAP assay kit was purchased from Beyotime Biotechnology (Shanghai, China). Loquat flowers of Baiyu were gathered in the Dongshan Zhen, Suzhou, Jiangsu Province, China during the winter. All flowers were dried in a drying oven (DHG-9055A; Yiheng, China) until the constant weight followed by powdering in a cutting mill (FW100; Tianjin Taisite Instrument, China). The obtained powder was passed through 60 mesh sieve and kept before analysis at −20°C.

### 2.2 Selection of extraction conditions

The 2.0 g of powdered flower material was extracted under ultrasonic conditions for phenolics. Experimental values used as of the independent variables in experimental design are represented in Supplementary Table 1. The powder was added to a container after the fixed temperature was reached. The constant values for solid–liquid ratio, EtOH concentration, extraction temperature, and duration of the experiment were 1:50 (g:mL), 50%, 60°C, and 20 min, respectively. All experiments were repeated three times independently.

### 2.3 Response surface method (RSM) for finding the optimal extraction conditions

Box-Behnken design (BBD) was utilized to examine the influence of different experimental parameters on the efficiency of crude total polyphenolics (CTP). In this experiment, four variables were evaluated at three levels (low, middle, and high level). Overall, 29 repeated experiments at various experimental conditions were done, taking the extraction yield of total phenolics (TP) and total flavonoids (TF) as a response. The interactions between pairs of variables were evaluated from the surface plots.

### 2.4 Quantification of total phenolics and flavonoids

The TP content was determined using the Folin-Ciocalteu (F-C) procedure. As an illustration, 0.1 mL of extract was mixed with 3.9 mL dH_2_O, mixed, 1 mL of F-C reagent, and 5 ml of Na_2_CO_3_ (15%, w/v) solutions were added sequentially. After 30 min incubation at room temperature (RT), the absorbance was measured at 760 nm (TU 1900 spectrophotometer, PERSEE Bio. Tec., Beijing, China). The TF content was determined by mixing 0.1 mL of extract, 5.3 mL dH_2_O, and 0.3 mL NaNO_2_ (5%, w/v). The mixture was stirred at RT for 5 min, followed by the addition of 0.3 mL Al(NO_3_)_3_ (10%, w/v). After 6 min, 4 mL of NaOH (4%, w/v) solution was added, and A_510_ was recorded 15 min after the incubation.

### 2.5 Antioxidant activity assay

To evaluate the antioxidant capacities of TP, ABTS, DPPH, and FRAP assays were conducted. For ABTS assay, a sample (100 μL) was added to ABTS (3.9 mL). After 6 min, the absorbance was measured at 734 nm. For DPPH assay, a sample (100 μL) was added to DPPH (3 mL) methanolic solution (0.1 mol/L). After 30 min, the absorbance was measured at 517 nm using a spectrophotometer. For FRAP assay, 3 ml of FRAP reagent, prepared freshly, was mixed with 100 μL of the sample. The absorbance of the reaction mixture at 593 nm was measured spectrophotometrically after incubation at 25°C for 5 min. ABTS, DPPH, and FRAP values were reported relative to ascorbic acid (AA), in mg AA equivalent/g dry weight (mg AAE/d.w.).

### 2.6 Enzyme activity assay

The inhibitory activity of PPO toward diphenolase was investigated, taking L-DOPA as substrate. In a 3 mL solution, 0.5 mM of L-DOPA in 50 mM phosphate buffer (pH 6.8) was added along with 0.1 mL of the increasing amounts of PTP in DMSO, up to 3.33 μg/mL. The increased A_475_ value with molar absorption coefficient ϵ = 3700 M^−1^ cm^−1^ recorded on a Beckman UV-800 spectrophotometer was used to monitor an enzyme activity. The reaction was carried out at 30°C.

### 2.7 Scanning study

The L-DOPA oxidation was conducted with and without the addition of PTP. The reaction solution (3 mL) consisted of 0.5 mM L-DOPA in 50 mM PBS buffer (pH 6.8) and 0.1 mL of DMSO solution of PTP. The amount of PPO was 16.67 μg/mL. The reaction was monitored spectrophotometrically (TU 1900, PERSEE Bio. Tec., Beijing, China).

### 2.8 Molecular docking model

Molecular operation environment software (MOE) is often used for protein-ligand docking. The refinement was set to the force field, the retention of scoring was set to 20 and ranked by London dG, and the other parameters used were set as default.

### 2.9 Statistical analysis

GraphPad Prism 6 (San Diego, CA, USA) and Design Expert 8.0 (Minneapolis, MN, USA) was used for analysis with data of three independent experiments expressed as means ± SEM. For comparing the two groups, One-way ANOVA was applied. The data at the statistical level P* ≤ 0.05 were taken as significant.

## 3. Results

### 3.1 The influence of extraction variables on the extraction yield of TP

**Figure 1.**
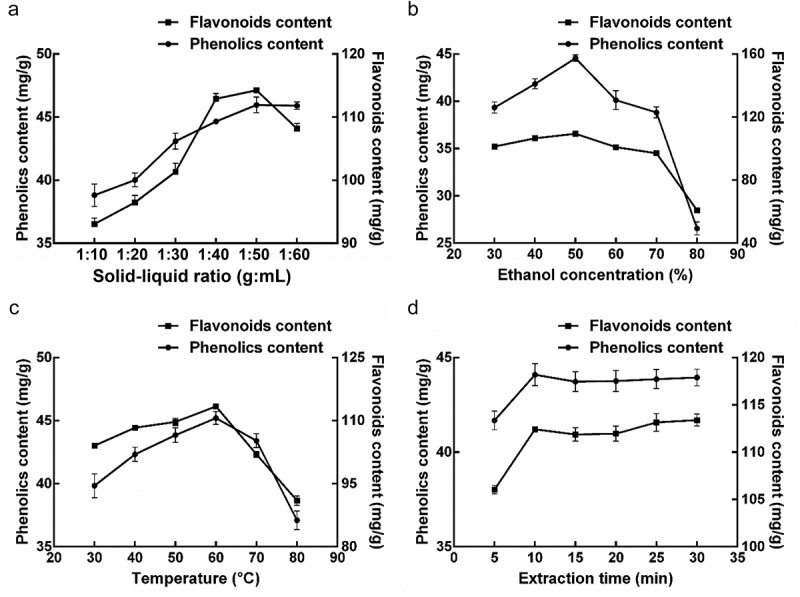
The influence of solid to liquid ratio (a), EtOH concentration (b), temperature (c), and time (d) on TP and TF extraction from loquat flowers.

The influence of four experimental variables, i.e. solid to liquid ratio, EtOH concentration, extraction temperature, and duration on the extraction efficiency of TP from loquat flower, were explored by a single-factor method. Generally, the more solvent volume dissolves TP more effectively and results in a higher extraction yield. The result indicates that the yield significantly increased when the ratio of solid to liquid decreased from 1:10 to 1:50 g/mL; after 1:50 ratio, the yield of TP declined slightly (Figure 1a). Therefore, 1:50 ratio was enough for extraction. The impact of EtOH concentration on the extraction efficiency of TP is illustrated in Figure 1b. Increasing the EtOH concentration from 30% to 50% (v/v) the extraction yield was improved, while concentrations above 50% diminished the extraction efficiency. Therefore, the preferred EtOH concentration was 50%. The extraction efficiency of TP increased when the temperature rose from 30°C to 60°C and decreased when the temperature was above 60°C (Figure 1c). The influence of the duration of extraction on the extraction efficiency of TP was demonstrated. Prolonging the duration of extraction from 5 to 10 min significantly increased the efficiency of extraction, while the yield was almost unchanged from 10 to 30 min (Figure 1d). In summary, the optimum values of extraction parameters were: solid to liquid ratio 1:50 g/mL, EtOH concentration 50%, temperature 60°C and time 10 min.

### 3.2 Optimization of TP extraction by RSM

**Figure 2.**
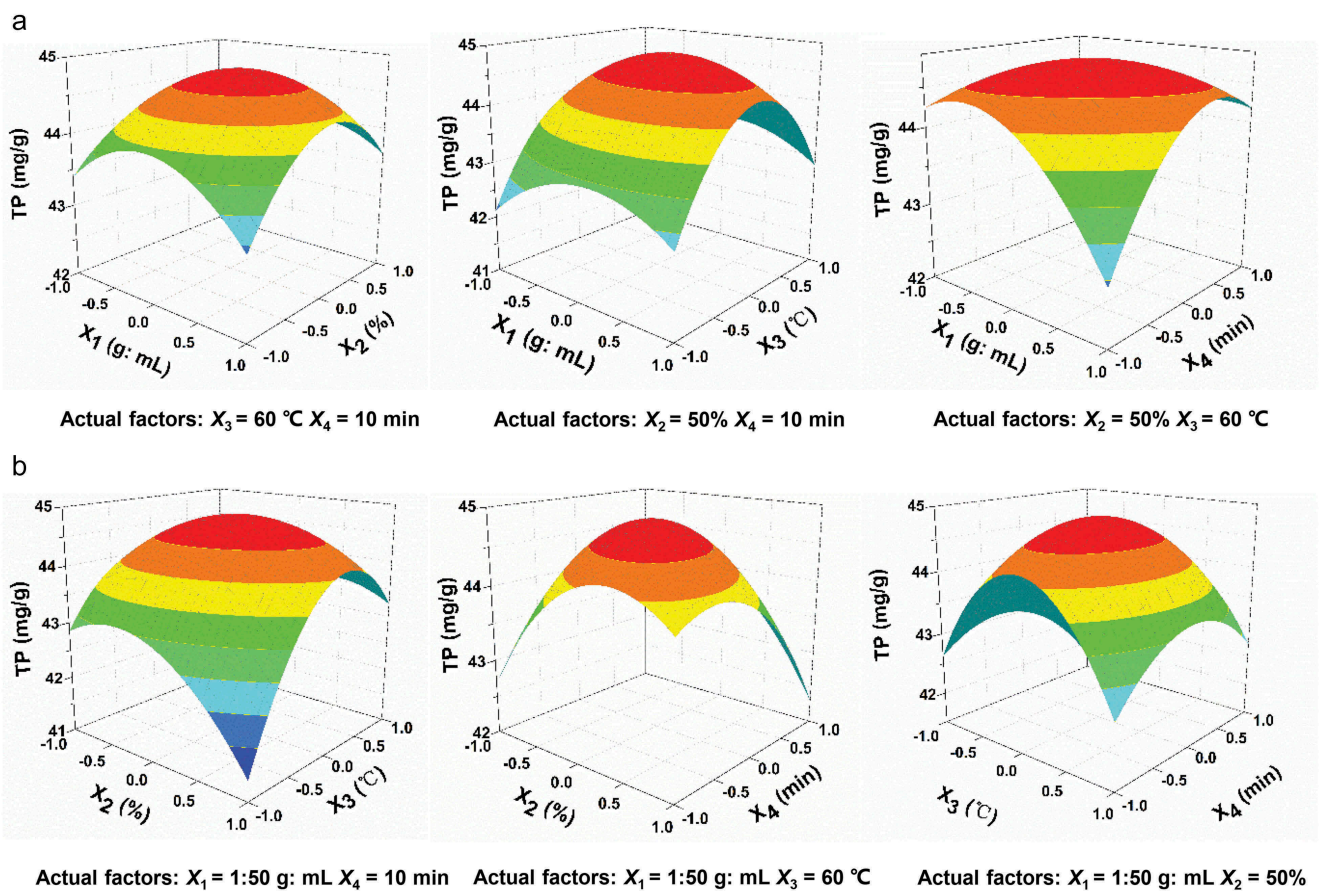
Three-dimensional response surface contour plots showing the effect of co-variance in solid to liquid ratio (*X*_1_)/EtOH concentration (*X*_2_) (a, left), solid to liquid ratio (*X*_1_)/temperature (*X*_3_) (a, middle), solid to liquid ratio (*X*_1_)/time (*X*_4_) (a, right), EtOH concentration (*X*_2_)/temperature (*X*_3_) (b, left), EtOH concentration (*X*_2_)/time (*X*_4_) (b, middle), and temperature (*X*_3_)/time (*X*_4_) (b, right) on TP from loquat flower.

Based on the results of single-factor experiments, RSM was used to optimize the experimental conditions. The underlying mathematical model and the optimized values of all experimental conditions for the extraction were obtained using Design Expert 8.0 software. Four experimental factors, previously evaluated in single-factor experiments (Table S1), were selected for optimization (Table S2). All examined values of four independent variables (*X*_1_, *X*_2_, *X*_3_ and *X*_4_) along with the obtained and predicted values for dependent variables (responses, TP and TF) are listed in Table S3. An excellent correlation observed for the experimental and modeled response values demonstrated a high predictivity of the developed mathematical model. The ideal extraction conditions obtained for TP and TF were solid to liquid ratio 1:50 g/mL and 1:51 g/mL, ethanol concentration 50% and 51%, temperature 61°C and 58°C, and time 9 min and 10 min, respectively (Table S4). The effect of simultaneous variation of each of the four experimental variables could be visualized from the trends shown in the form of 3D plots (Figure 2 and Figure S1).

### 3.3 Antioxidant activity of TP from loquat flower

**Figure 3.**
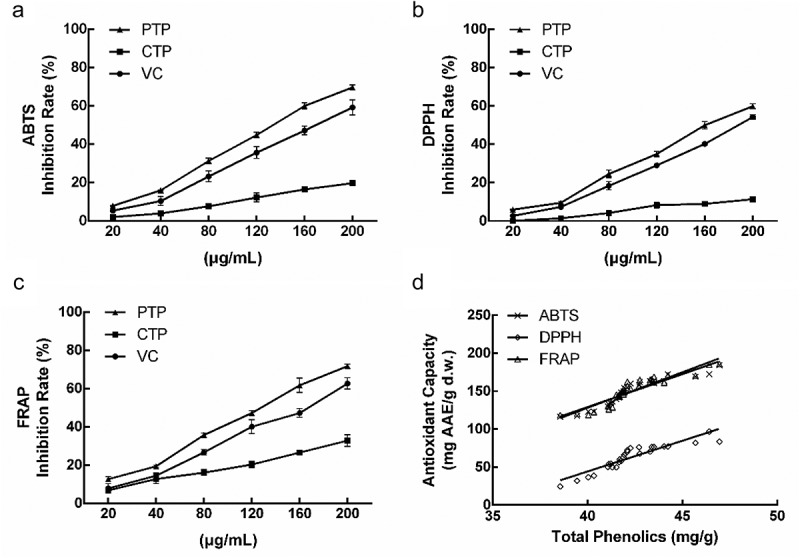
Antioxidant capacity obtained from three different assays, ABTS (a), DPPH (b), and FRAP (c). Correlation analysis between phenolics and antioxidant capacity (d).

Considering the complexity of TP in natural products and diverse antioxidant reaction-mechanism assays, commonly ABTS, DPPH and FRAP assays were performed to assess the antioxidant capacity of TP. The antioxidant capacity of purified total phenolics (PTP) was higher than VC, and the values on *IC*_50_ are 126.3 ± 8.9, 162.4 ± 6.3 and 94.97 ± 6.6 mg AAE/d.w., respectively (Figure 3a–c). According to correlation analyses (Figure3d and Table S6), the TP of loquat flower exhibited strong correlations with ABTS (*R*^2^ = 0.866, *P* < 0.001), DPPH (*R*^2^ = 0.833, *P* < 0.0001) and FRAP (*R*^2^ = 0.857, *P* < 0.001).

### 3.4 The inhibitory activity of PTP on PPO

**Figure 4.**
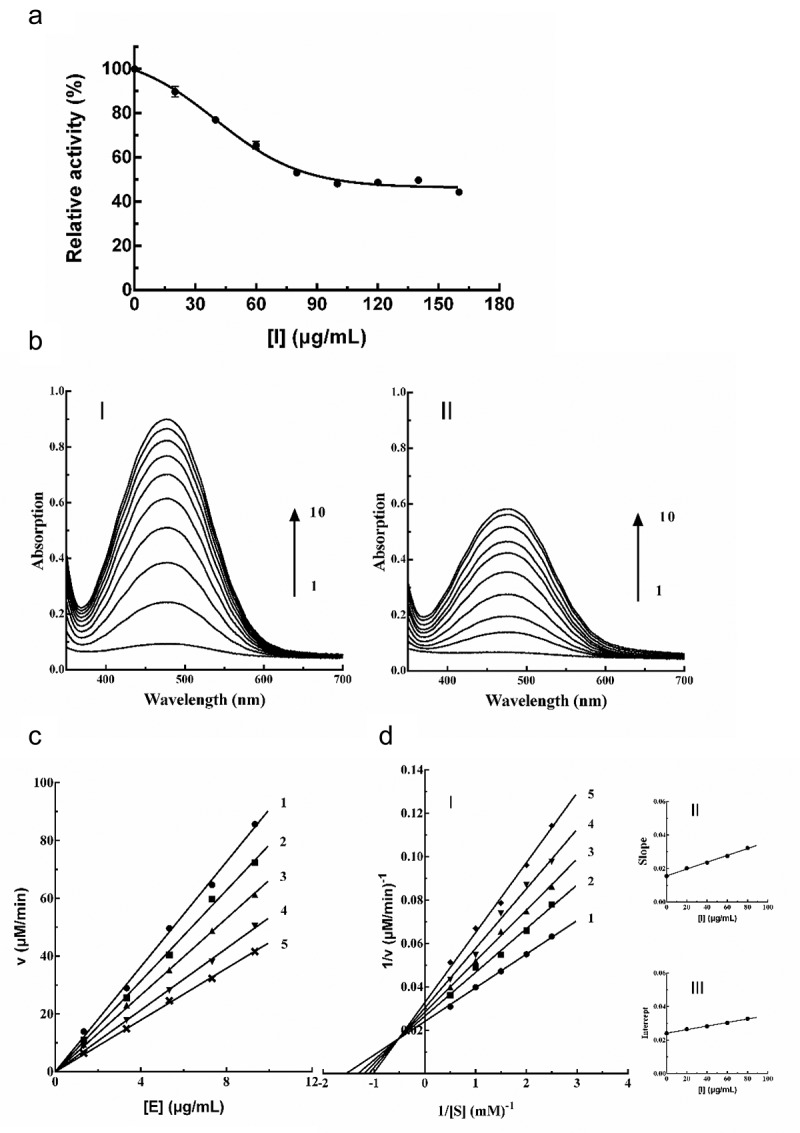
Inhibitory effects of PTP toward PPO (a). Consecutive spectra obtained during the PPO catalysis with (B I) and without PTP (100 μg/mL, B II), where lines 1–10 indicate 0–9 min upon the addition of PPO. Inhibitory mechanism of PTP toward PPO (c), and type of inhibition (d); for line 1–5 the corresponding PTP concentrations were 0, 20, 40, 60, 80 μg/mL, respectively.

The influence of PTP on PPO activity was evaluated. The PTP inhibited the PPO activity in a dose-dependent fashion (Figure 4a). The corresponding *IC*_50_ of PTP on PPO was 115 ± 9.2 μg/mL.

### 3.5 Full wavelength scanning for the product in the presence and absence of PTP

The oxidation of L-DOPA by PPO was monitored spectrophotometrically in the absence (Figure 4b I) and presence (Figure 4b II) of PTP (60 μg/mL). In the presence of PTP, 9 min upon addition of PPO a typical A_475_ peak decreased by 35% (from 0.90 to 0.58).

### 3.6 Inhibition mechanism of PTP activity toward PPO

The underlying mechanism of how PTP inhibits PPO activity was studied. The slope of the line decreased with rising concentrations of PTP, and several straight lines all intersect y-axis in the origin (Figure 4c), suggesting the reversible inhibition of PPO by PTP. It can be concluded that the presence of PTP did not reduce the amount of active enzyme, but just led to a decrease in the reaction activity of the enzyme.

### 3.7 Inhibition type of PTP toward PPO

Lineweaver–Burk plots for PTP to PPO in terms of 1/v *vs*. 1/[S] produced a family of lines converging to a point at the second quadrant (Figure 4d), indicating that the PTP from loquat flower were mixed-type inhibitors. This indicated that the inhibitor could bind not only with the free enzyme but also with the enzyme-substrate complex, and their equilibrium constants were different. The equilibrium constants for binding of PTP to enzyme (*K*_I_ = 76.77 μg/mL) and the enzyme-substate complex (*K*_IS_ = 227.86 μg/mL) were calculated from the plots of slope/intercept *vs*. the amount of PTP. The value of *K*_IS_ was higher than *K*_I_, which indicated that the combination of PTP and free enzyme was stronger than that between the compounds and enzyme-substrate complexes.

### 3.8 Molecular docking analysis

**Figure 5.**
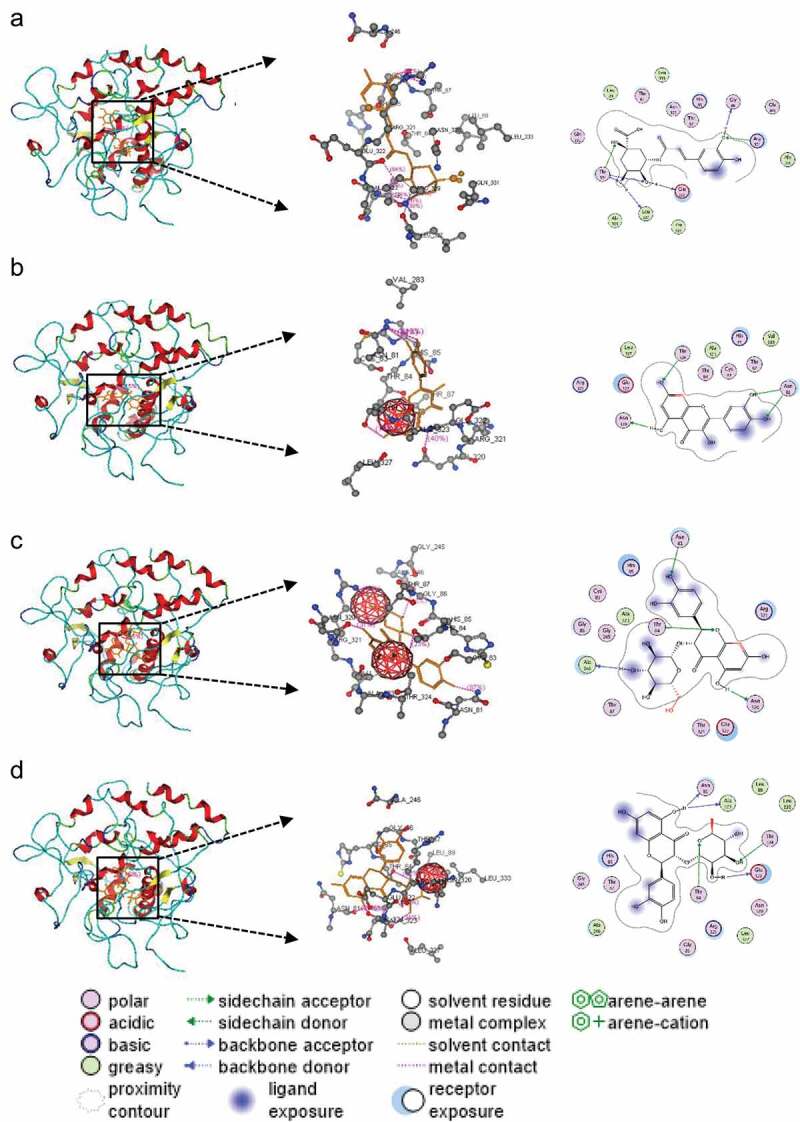
Docking of chlorogenic acid (a), quercetin (b), isoquercetin (c), and quercitrin (d) ligands with PPO residues.

To explain the effects of the main units of PTP on the activity of PPO, we further examined the docking mode of PTP units in the catalytic site of PPO. Figure 5 depicts the docked conformation of the main units of PTP: chlorogenic acid, quercetin, isoquercetin, and quercitrin in the PPO catalytic center. The intermolecular forces between chlorogenic acid, quercetin, isoquercetin or quercitrin, and PPO mainly were hydrogen bonds and hydrophobic interactions. Chlorogenic acid could form interactions with PPO residues: Thr84, His85, Gly86, Leu89, Gly245, Ala246, Glu322, Ala323, Thr324, Leu327, Pro329, Gln331, and Leu333 (Figure 5a). Quercetin could form interactions with PPO residues: Asn81, Gys83, Thr84, His85, Thr87, Val283, Asn320, Arg321, Glu322, Ala323, Thr324, and Leu327 (Figure 5b). Isoquercetin could form interactions with PPO residues: Asn81, Gys83, Thr84, Gly86, Thr87, Gly245, Ala246, Asn320, Glu322, Ala323, and Thr324 (Figure 5c). Quercitrin could form interactions with PPO residues: Asn81, Thr84, His85, Gly86, Thr87, Leu89, Gly245, Ala246, Asn320, Glu322, Ala323, Thr324, Leu327, and Leu333 (Figure 5d). These findings were in accordance with the results of dynamic analysis and could provide a viable intrinsic mechanism to understand the efficient inhibition of PTP on PPO.

## 4. Discussion

Polyphenolic compounds from plants received considerable attention with regards to exhibiting diverse bioactivity and provide versatile health benefits to humans, including antioxidant, anti–inflammatory, and anticancer activity [14]. In our present work, we optimized the extraction conditions of polyphenolic from loquat, and the solid to liquid ratio, EtOH concentration, temperature, and duration of extraction were 1:50 g/mL, 50%, 61°C, and 9 min, respectively, which constitutes to the optimum conditions. To our knowledge, this is the first demonstration of the optimum condition of polyphenolic from loquat flowers by RSM.

Natural polyphenolics studied for their potent use in treating various diseases [15–19], which attributes the success to the high antioxidant activity of polyphenolics. Our study showed that the antioxidant activity of polyphenolics from the loquat flower was strong, and the antioxidant potency was positively correlated with TP content. In addition, the inhibitory effect of PTP toward PPO activity was demonstrated with an *IC*_50_ 115 ± 9.2 μg/mL. The double-reciprocal plots of PPO in the presence of PTP revealed a mixed-type inhibition. The *K*_I_ value for PTP-PPO binding was higher than *K*_IS_, demonstrating that the higher affinity of PTP toward PPO-substrate complexes that to the free PPO [20].

## 5. Practical applications

PTP was proven to be a good antioxidant and exhibited an extremely effective inhibitory effect on PPO activity. It was proved to be a reversible and mixed-type inhibitor of the enzyme. Therefore, this study confirmed a new and efficient PPO inhibitor, which laid a scientific foundation for the feasible usage of PTP in the field of the food industry.

## 6. Conclusions

In summary, this study demonstrated that the ideal conditions for the extractions of polyphenolics from loquat flowers were solid to liquid ratio 1:50 g/mL, ethanol concentration 50%, temperature 61°C, and time 9 min, respectively. Furthermore, PTP exhibited high levels of antioxidant capacity and inhibitory activity on PPO. PTP was reversible and mixed inhibitors of PPO. These findings establish a scientific basis in the screening for PPO inhibitors. In addition, PTP were potent antioxidants. The results of this study revealed that PTP from loquat flowers have possible applicability in the food industry.

## Highlights

- The conditions of extraction of loquat flowers polyphenolics were optimized.
- Purified total polyphenolics (PTP) displayed strong antioxidant activity.
- PTP is efficient, reversible, and mixed competitive inhibitors of PPO.

## Supplementary Material

The supplementary data for this article can be accessed here.
