# Supplementary material for: Optimization of extraction of loquat flowers polyphenolics and its antioxidant and anti-polyphenol oxidase properties
Source: Bioengineered. 2020 Mar 3;11(1):281–90. doi: 10.1080/21655979.2020.1735604 (PMC7161536; doi:10.1080/21655979.2020.1735604)
Supplement: Supplemental Material [file kbie-11-01-1735604-s001.docx]

Supplementary Fig. 1


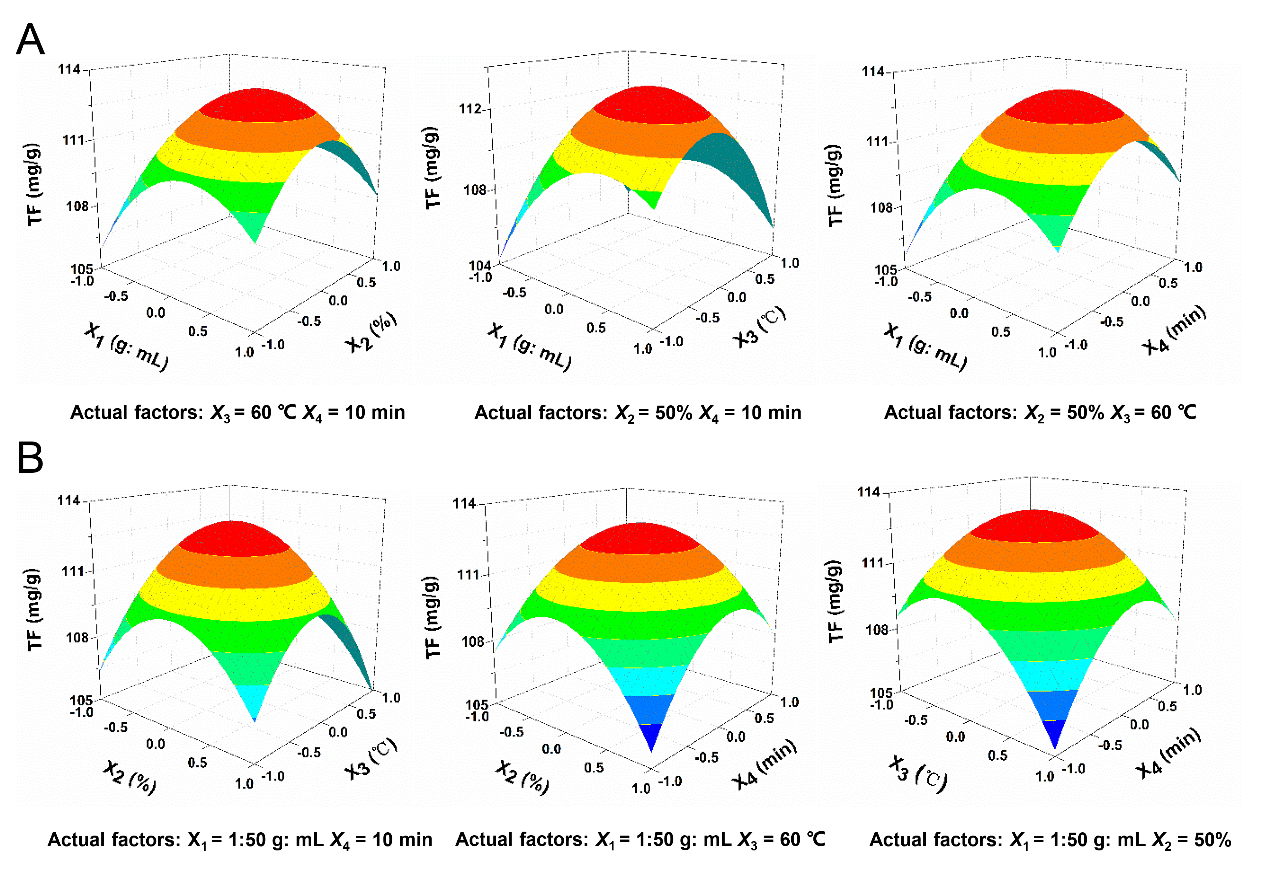


**Supplementary Fig. 1** Three-dimensional response surface contour plots showing the effect of co-variance in solid to liquid ratio (*X*_1_)/EtOH concentration (*X*_2_)(A, left), solid to liquid ratio (*X*_1_)/temperature (*X*_3_) (A, middle), solid to liquid ratio (*X*_1_)/time (*X*_4_) (A, right), EtOH concentration (*X*_2_)/temperature (*X*_3_) (B, left), EtOH concentration (*X*_2_)/time (*X*_4_) (B, middle), and temperature (*X*_3_)/time (*X*_4_) (B, right). on TF from loquat flower.

Supplementary Tables

**Table S1:** Experimental values of the independent variables for the single-factor experiment

| Independent variables | Experimental values | | | | | |
| --- | --- | --- | --- | --- | --- | --- |
| *r*_SL_ (g: mL) | 1:10 | 1:20 | 1:30 | 1:40 | 1:50 | 1:60 |
| *C*_e_ (%) | 30 | 40 | 50 | 60 | 70 | 80 |
| *T*_e_ (°C) | 30 | 40 | 50 | 60 | 70 | 80 |
| *t*_e_ (min) | 5 | 10 | 15 | 20 | 25 | 30 |

*r*_SL:_ solid-liquid ratio; *C*_e_: ethanol concentration; *T*e: extraction temperature; *t*_e_: extraction time.

**Table S2** Experimental values and coded levels of the independent variables used for the 29-full factorial design

| Independent variables | Code units | Experimental values | | |
| --- | --- | --- | --- | --- |
|  |  | -1 | 0 | 1 |
| *r*_SL_ (g: mL) | *X*_1_ | 1:40  40  50  5 | 1:50 | 1:60 |
| *C*_e_ (%) | *X*_2_ |  | 50 | 60 |
| *T*_e_ (°C) | *X*_3_ |  | 60 | 70 |
| *t*_e_ (min) | *X*_4_ |  | 10 | 15 |

*r*_SL:_ solid-liquid ratio; *C*_e_: ethanol concentration; *T*e: extraction temperature; *t*_e_: extraction time.

**Table S3 Plan and results for response surface methodology of TP and TF**

|  | Independent variable | | | |  | Response (mg/g) | | | |
| --- | --- | --- | --- | --- | --- | --- | --- | --- | --- |
| Run | *X*_1_  (g:mL) | *X*_2_  (%) | *X*_3_  (°C) | *X*_4_  (min) | Actual  TP | | Predicted  TP | Actual  TF | Predicted  TF |
| 1 | 1:60 (+1) | 50 (0) | 60 (0) | 15 (+1) | 44.12 | | 44.18 | 108.95 | 108.81 |
| 2 | 1:40 (-1) | 50 (0) | 60 (0) | 15 (+1) | 42.12 | | 42.09 | 106.48 | 106.40 |
| 3 | 1:50 (0) | 40 (-1) | 50 (-1) | 10 (0) | 42.57 | | 42.84 | 106.44 | 106.31 |
| 4 | 1:60 (+1) | 60 (+1) | 60 (0) | 10 (0) | 43.64 | | 43.63 | 107.95 | 108.09 |
| 5 | 1:50 (0) | 60 (+1) | 60 (0) | 5 (-1) | 43.92 | | 43.91 | 105.54 | 105.65 |
| 6 | 1:60 (+1) | 50 (0) | 60 (0) | 5 (-1) | 42.60 | | 42.72 | 108.44 | 108.23 |
| 7 | 1:50 (0) | 50 (0) | 60 (0) | 10 (0) | 44.86 | | 44.84 | 113.15 | 113.10 |
| 8 | 1:60 (+1) | 50 (0) | 70 (+1) | 10 (0) | 42.80 | | 42.82 | 105.51 | 105.56 |
| 9 | 1:50 (0) | 40 (-1) | 60 (0) | 15 (+1) | 43.60 | | 43.60 | 106.57 | 106.59 |
| 10 | 1:40 (-1) | 50 (0) | 50 (-1) | 10 (0) | 42.15 | | 42.12 | 104.27 | 104.34 |
| 11 | 1:50 (0) | 40 (-1) | 60 (0) | 5 (-1) | 42.78 | | 42.68 | 107.26 | 107.40 |
| 12 | 1:50 (0) | 50 (0) | 60 (0) | 10 (0) | 44.82 | | 44.84 | 113.06 | 113.10 |
| 13 | 1:50 (0) | 50 (0) | 60 (0) | 10 (0) | 44.85 | | 44.84 | 113.18 | 113.10 |
| 14 | 1:50 (0) | 40 (-1) | 70 (+1) | 10 (0) | 41.80 | | 41.80 | 105.54 | 105.53 |
| 15 | 1:60 (+1) | 50 (0) | 60 (-1) | 10 (0) | 42.54 | | 42.43 | 109.17 | 109.34 |
| 16 | 1:50 (0) | 50 (0) | 60 (0) | 10 (0) | 44.81 | | 44.84 | 113.13 | 113.10 |
| 17 | 1:40 (-1) | 40 (-1) | 60 (0) | 10 (0) | 43.47 | | 43.39 | 105.75 | 105.76 |
| 18 | 1:40 (-1) | 50 (0) | 70 (+1) | 10 (0) | 42.48 | | 42.58 | 105.50 | 105.45 |
| 19 | 1:50 (0) | 60 (+1) | 60 (0) | 15 (+1) | 42.20 | | 42.29 | 107.91 | 107.90 |
| 20 | 1:50 (0) | 50 (0) | 50 (-1) | 5 (-1) | 42.72 | | 42.63 | 107.23 | 107.28 |
| 21 | 1:40 (-1) | 60 (+1) | 60 (0) | 10 (0) | 42.77 | | 42.77 | 105.58 | 105.75 |
| 22 | 1:50 (0) | 60 (+1) | 70 (+1) | 10 (0) | 43.40 | | 43.23 | 104.91 | 104.76 |
| 23 | 1:40 (-1) | 50 (0) | 60 (0) | 5(-1) | 44.21 | | 44.25 | 105.69 | 105.54 |
| 24 | 1:50 (0) | 50 (0) | 70 (+1) | 5 (-1) | 42.46 | | 42.50 | 104.00 | 104.05 |
| 25 | 1:50 (0) | 50 (0) | 60 (0) | 10 (0) | 44.87 | | 44.84 | 112.96 | 113.10 |
| 26 | 1:50 (0) | 60 (+1) | 50 (-1) | 10 (0) | 41.24 | | 41.34 | 106.92 | 106.65 |
| 27 | 1:50 (0) | 50 (0) | 70 (+1) | 15 (+1) | 42.69 | | 42.70 | 106.57 | 106.67 |
| 28 | 1:50 (0) | 50 (0) | 50 (-1) | 15 (+1) | 41.87 | | 41.73 | 106.00 | 106.10 |
| 29 | 1:60 (+1) | 40 (-1) | 60 (0) | 15 (0) | 43.18 | | 43.09 | 108.54 | 108.52 |

*X*_1:_ solid-liquid ratio; *X*_2_: ethanol concentration; *X*_3_: extraction temperature; *X*_4_: extraction time. The coded forms of the variables are shown in parentheses.

**Table S4 Ideal predicted conditions and theoretically calculated maximal values for TP and TF**

| Response | Maximum predicted value  (mg/g) | Ideal condition | | | | | |
| --- | --- | --- | --- | --- | --- | --- | --- |
|  |  | *r*_SL_ (g: mL) | *C*_e_ (%) | | *T*e (°C) | | *t*_e_ (min) |
| TP | 44.93 | 1:50 | | 50 | | 61 | 9 |
| TF | 113.30 | 1:51 | | 49 | | 58 | 10 |

*r*_SL:_ solid-liquid ratio; *C*_e_: ethanol concentration; *T*e: extraction temperature; *t*_e_: extraction time.

**Table S5 Correlation analysis between phenolics and antioxidant capacity**

| Antioxidant Capacity | TP | *R*^2^ | *P* |
| --- | --- | --- | --- |
| ABTS | *y* = 8.670*x* - 217.7 | 0.8658 | <0.0001 |
| DPPH | *y* = 8.090*x* - 279.4 | 0.8329 | <0.0001 |
| FRAP | *y* = 9.373*x* - 246.8 | 0.8567 | <0.0001 |
